# Supplementary material for: The role of GPT in promoting inclusive higher education for people with various learning disabilities: a review
Source: PeerJ Comput Sci. 2025 Feb 6;11:e2400. doi: 10.7717/peerj-cs.2400 (PMC11888875; doi:10.7717/peerj-cs.2400)
Supplement: Supplemental Information 1 [file peerj-cs-11-2400-s001.pdf]

Rebuttal Letter for Addition of Author to Manuscript Submission

Date: 21-05-2024

To the Editorial Board,

We are writing to provide justification for the addition of a new author, Dr. Yaodong Zhu, to our paper. Following the feedback received from the peer review, significant revisions and additional experiments were necessitated, to which Dr. Zhu has contributed substantially.

Dr. Zhu played a crucial role in addressing the reviewers' comments. His suggestions have filled critical gaps in our research, providing essential data that substantially enhance the manuscript's validity and impact. He has played a pivotal role in analyzing the data. His expertise and analysis have allowed us to extract meaningful insights, which have significantly strengthened our findings. He contributed to the rewriting of several sections of the manuscript. He has also assisted in revising the entire manuscript to improve its clarity and coherence, ensuring that the additions are well-integrated with the original content. He reviewed and approved the final manuscript as submitted, agreeing to take responsibility for all aspects of the work. All co-authors have agreed to his addition and have approved the final version of the manuscript. We believe that Dr. Zhu’s contributions are substantial and align with the authorship criteria outlined by PeerJ. His involvement directly responds to the need for additional expertise prompted by the initial peer reviews.

We appreciate the opportunity to revise our manuscript and trust that the contributions of Dr. Zhu justify his inclusion as an author. We look forward to your feedback and are hopeful for the positive progression of our submission.

Thank you for your consideration.

Sincerely,

Wei Wang and other authors
